# Dissipation and Residues of Dichlorprop-P and Bentazone in Wheat-Field Ecosystem

**DOI:** 10.3390/ijerph13060534

**Published:** 2016-05-26

**Authors:** Xiaoxiao Feng, Jianlei Yu, Lixiang Pan, Guochun Song, Hongyan Zhang

**Affiliations:** 1 College of Science, China Agricultural University, Beijing 100193, China; xxf0423@cau.edu.cn (X.F.); panlixiang5545@163.com (L.P.); 2 Institute of Plant Protection, Shandong Academy of Agricultural Sciences, Jinan 250100, China; jlyu2010@163.com (J.Y.); songguochun2008@163.com (G.S.)

**Keywords:** dichlorprop-P, bentazone, dissipation, residue, wheat

## Abstract

Dichlorprop-P and bentazone have been widely used in the prevention and control of weeds in wheat field ecosystems. There is a concern that pesticide residues and metabolites remain on or in the wheat. Thus, the study of the determination and monitoring of their residues in wheat has important significance. A rapid, simple and reliable QuEChERS (Quick, Easy, Cheap, Effective, Rugged and Safe) method was modified, developed and validated for the determination of dichlorprop-P, bentazone and its metabolites (6-hydroxy-bentazone and 8-hydroxy-bentazone) in wheat (wheat plants, wheat straw and grains of wheat) using high-performance liquid chromatography coupled with tandem mass spectrometry (HPLC-MS/MS). The average recoveries of this method ranged from 72.9% to 108.7%, and the limits of quantification (LOQs) were 2.5–12 μg/kg. The dissipation and final residue of four compounds in three provinces (Shandong, Jiangsu and Heilongjiang) in China were studied. The trial results showed that the half-lives of dichlorprop-P and bentazone were 1.9–2.5 days and 0.5–2.4 days in wheat plants, respectively. The terminal residues in grains of wheat and wheat straw at harvest were all much below the maximum residue limit (MRL) of 0.2 mg/kg for dichlorprop-P and 0.1 mg/kg for bentazone established by the European Union (EU, Regulation No. 396/2005).

## 1. Introduction

Wheat, the leading source of vegetable protein in human food, is one of the most important food crops in the world, but the impact of field weeds, plant diseases and pests on wheat production is a big issue. To control these negative factors, pesticides play an important role, being introduced on a regular basis [1]. Among those, bentazone and dichlorprop-P have been applied to protect wheat from weeds.

Bentazone, 3-(1-Methylethyl)-1H-2, 1, 3-benzothiadiazin-4(3H)-one-2, 2-dioxide, is a diazosulfide herbicide of broad spectrum, effective and low toxicity. It has been used to control broadleaf weeds and nutgrass flatsedge in a variety of crops. For example, bentazone is applied on wheat to prevent buglossoides arvense and galium aparine [2]. Bentazone is a fatty acid synthesis inhibitor that works via the inhibition of acetyl CoA carboxylase [3]. In plants, two derivatives hydroxylated at the 6 and 8 position of the aromatic ring are found besides the unchanged parent compound. In addition, 6-hydroxy-bentazone and 8-hydroxy-bentazone are included in the definition of bentazone residues for compliance with the Maximum Residue Limit (MRL), which was reported by the Joint Meeting on Pesticide Residues (JMPR) [4], the Codex Alimentarius Commission (CAC) [5] and the European Union (EU) [6]. China [7], the CAC [5] and the EU (Regulation No. 396/2005) [6] all legislated that the MRL of bentazone was 0.1 mg/kg.

**Figure 1:**
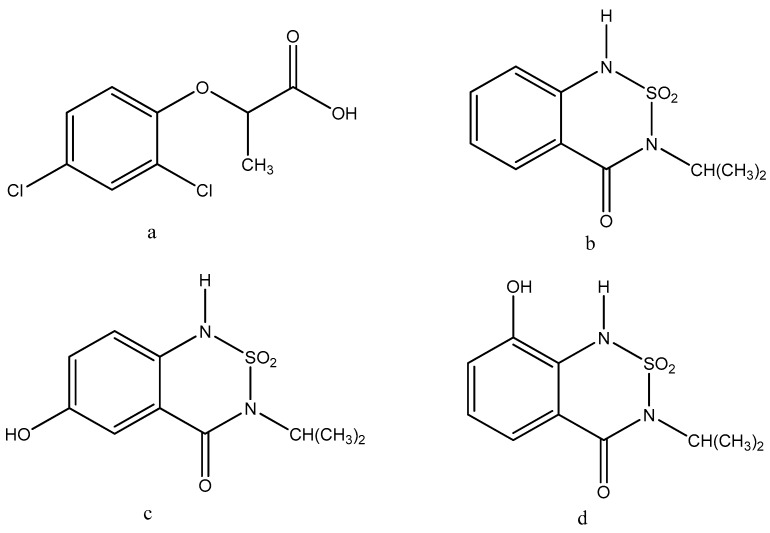
Chemical structures of (**a**) dichlorprop-P; (**b**) bentazone; (**c**) 6-hydroxy-bentazone and; (**d**) 8-hydroxy-bentazon.

Dichlorprop-P (2,4-dichlorophenoxypropanoic acid, or 2,4-DP), a component of many common weed killers, is widely used for the control of broadleaf weeds, annual and perennial weeds in cereals, pastures and forestry [8]. The residue definition for the dichlorprop established by the EU (Regulation No. 396/2005) was the sum of dichlorprop (including dichlorp-P) and its conjugates, with the MRL of dichlorprop-P in wheat being 0.2 mg/kg [6]. There is a growing awareness and concern that some pesticide residues and metabolites remaining on or in the harvested crop could be edible [9]. Therefore, it is necessary to establish a reliable and sensitive simultaneous determination procedure for dichlorprop-P, bentazone and its metabolites (6-hydroxy-bentazone and 8-hydroxy-bentazone) to ensure public health. The chemical structures of four analytes are shown in Figure 1.

In recent years, some sample preparation methods have been reported, with solid phase extraction (SPE) [10,11,12,13], liquid-liquid extraction [14], supercritical fluid extraction (SFE) [15,16], and gel permeation chromatograph (GPC) [17] being widely adopted for the detection of bentazone and dichlorprop-P. However, most of them reported the determination of bentazone and dichlorprop-P, but few papers covered the analysis of 6-hydroxy-bentazone and 8-hydroxy-bentazone. Moreover, these methods are tedious, complicated and expensive due to the use of a large amount of organic solvent, a series of steps for cleanup or preconcentration. In many studies, bentazone requires derivatization (e.g., with pentafluorobenzyl bromide (PFBBr) [12,18,19], diazomethane [20,21], and so on) prior to analysis. The derivatization needs a fair amount of sample manipulation and is time-consuming, which affects the progress of residual experiments to a certain extent. Recently, the Quick, Easy, Cheap, Effective, Rugged and Safe (QuEChERS) method introduced by Anastassiades [22] has been an attractive alternative method for sample preparation. It is based on an extraction with organic solvent followed by a partitioning step and then cleaning up by dispersive solid-phase extraction (d-SPE) [23]. The QuEChERS multiresidue procedure replaces or omits some complex analysis steps commonly employed in traditional methods. The QuEChERS method now serves as a template for sample preparation, which could be modified depending on the analytes properties, matrix composition, equipment and analytical technique available in the laboratory.

Liquid chromatography coupled with tandem mass spectrometry (HPLC-MS/MS) has become more and more popular for multiresidue analysis because of its high specificity and excellent separation for the target compounds. HPLC–MS/MS with triple quadrupole instruments in multiple reaction monitoring (MRM) mode is the traditional method to increase selectivity and improve sensitivity [24]. It also has been proven that HPLC–MS/MS in combination with QuEChERS is a rapid, highly sensitive and efficient method for the determination of pesticide residues [25,26].

The dissipation rate of a pesticide after application is a useful tool to evaluate the behavior of its residues [27]. Moreover, a preharvest interval (PHI) is required by MRL regulations to ensure the dissipation of a pesticide below the proposed MRL at harvest [28]. With the increasing use of dichlorprop-P and bentazone in wheat, the study of determination and monitoring of their residues in wheat has important significance. However, to the best of our knowledge and based on the available literature, there are no dissipation and residue studies of dichlorprop-P and bentazone in wheat.

The objective of the study was based on the QuEChERS method and was to develop and validate a rapid method for the analysis of dichlorprop-P, bentazone, 6-hydroxy-bentazone and 8-hydroxy-bentazone in wheat (wheat plants, wheat straw and grains of wheat). The dissipation dynamics of dichlorprop-P and bentazone in wheat plants were also studied, as well as the terminal residue in wheat straw and grains of wheat.

## 2. Materials and Methods

### 2.1. Materials and Reagents

Standard bentazone (purity was 99.7%), 6-hydroxy-bentazone (purity was 99.2%), 8-hydroxy-bentazone (purity was 99.5%), and dichlorprop-P (purity was 99.7%) were purchased from Ehrenstorfer GmbH, Augsburg, Germany; The individual stock standard solutions (1000 mg/L) of four analytes were dissolved with acetonitrile and stored at −20 °C, respectively. The individual work standard solutions (100 mg/L) were also prepared for daily use and stored at −20 °C. Acetonitrile was supplied by Fisher Scientific (Pittsburgh, PA, USA) in HPLC grade. Formic acid (98% purity) was purchased from Sinopharm Chemical Reagent Co. Ltd., Shanghai, China. Anhydrous magnesium sulfate (MgSO_4_) and sodium chloride (NaCl) purchased from Beijing Reagent Company (Beijing, China) were baked at 110 °C for 8 h. The Agela Cleanert C_18_ (40–60 μm), Primary secondary amine (PSA) and graphitized carbon black (GCB) were from Agela Technologies, Tianjin, China. The formulation, 566 g/L dichlorprop-P bentazone soluble liquid (SL), was kindly provided by BASF, Beijing, China. The 0.1% formic acid water solution was prepared before sample extraction. Ultrapure water was obtained from Aquapro Ultrapure Water System (Aquapro International Company LLC, Chongqing, China).

### 2.2. Field Trials

The field experiments, including the dissipation and residue experiments, were carried out at three different locations, Shandong Province (North China, the temperate continental monsoon climate), Jiangsu Province (East China, the subtropical monsoon climate) and Heilongjiang Province (Northeast China, the temperate continental monsoon climate) in the year 2015 according to the “Guideline on Pesticide Residue Trials”, published by the Ministry of Agriculture, People’s Republic of China (NY/T 788–2004).

There were four treatments including three dichlorprop-P bentazone treatments and one control treatment. The design of the field experiments for residue and dissipation was shown in Table 1. Each treatment consisted of three replicate plots and each experimental plot was 30 m^2^. No pesticide was used during the whole period of wheat growth in the control treatment. The buffer area of 30 m^2^ was used to separate the plots of different treatments.

In order to investigate the dissipation of dichlorprop-P and bentazone in wheat plants, the formulation of dichlorprop-P·bentazone (566 g/L) (SL) was dissolved in water and sprayed with a JACTO-HD400 internal pump backpack sprayer at active constituent 3820.5 g a.i./ha (gram of active gradient per hectare, 1.5 times of the recommended high dosage) on the stems and leaves of wheat. The experiment was carried out when weeds were at the 2–5 leaf stage. About 1 kg of wheat plant samples were collected randomly from several points in each plot at 2 h, 1, 2, 3, 5, 7, 10, 14, 21 and 30 days after spraying. The final residue experiments were performed at two dosage levels with 2547 g a.i./ha (recommended high dosage) and 3820.5 g a.i./ha (1.5 times recommended high dosage). Both the low and high dosage treatments were each sprayed one time when weeds were at 2–5 leaf stage. About 1.0 kg of wheat straw samples and 2.0 kg of grains of wheat were collected randomly from several points in each plot at the harvest time. Collected samples of wheat plants and wheat straw (1.0 kg) were cut into small pieces using a knife and were homogenized with a blender (Philips, Shanghai, China). Samples of grains of wheat (2.0 kg) were crushed with a high-speed grinder (Jiawei Instrument, Guangzhou, China). All samples were stored in a deep freezer at −20 °C until analysis within 2 months.

### 2.3. Sample Pretreatment

Frozen wheat samples were thawed at room temperature. Grains of wheat (5.0 g), wheat plants (2.0 g) and wheat straw (2.0 g) samples were weighed into a 50 mL plastic centrifuge tube. Then, 5 mL formic acid water (0.1%, *v*/*v*), 10 mL acetonitrile and 3.0 g NaCl were added into the tubes, and samples were subsequently extracted with a vortex mixer for 2 min and centrifuged for 5 min at 3800 rpm.

The supernatant acetonitrile layer of 1 mL was transferred into a 2 mL centrifuge tube with 100 mg anhydrous MgSO_4_ and vortexed for 30 s, then centrifuged at a relative centrifugal force (RCF) of 9168× *g* for 1 min. The upper layer was filtered through a 0.22 μm organic filter membrane to an auto-sampler vial for HPLC-MS/MS analysis.

### 2.4. HPLC-MS/MS Conditions

The HPLC–MS/MS analysis was achieved using an Agilent 1200 HPLC series (Agilent Technologies, Santa Clara, CA, USA) and an Agilent 6410B triple-quadrupole mass spectrometer equipped with an electrospray ionization interface (ESI±). A HPLC reverse-phase C_18_ column (50 mm × 2.1 mm × 3.5 μm, Agilent, Santa Clara, CA, USA) was employed for the separation of dichlorprop-P, bentazone, 6-hydroxy-bentazone and 8-hydroxy-bentazone at 30 °C. The mobile phase was acetonitrile and 0.1% formic acid water (*v*/*v* = 90/10) at a flow rate of 0.25 mL/min, and the injection volume was 5 μL. The total run time was 2.5 min. The HPLC–MS/MS was performed in negative multiple-reaction monitoring (MRM) mode. The desolvation gas (N_2_) temperature was set at 350 °C with the gas flow at 8.0 L/min, and the nebulizer pressure at 35 psi. The parameters were optimized individually for each target compound (Table 2).

### 2.5. Statistical Analysis

The dissipation patterns of dichlorprop-P and bentazone in the wheat plants were fitted to the first-order kinetics equation

C = C_0_e^−kt^ (1)

and half-life of residue was calculated by

*t*_1/2_ = (ln 2)/k (2)

where *t* is the time (day) after pesticide application, C represents the concentration (mg/kg) of the pesticide residue at the time of *t*, C_0_ represents the initial concentration (mg/kg) and k is the degradation rate constant in day^−1^. t_1/2_ is defined as the time required for the pesticide residue level to fall to half of the initial residue level after application. Systat Sigmaplot v12.0 software (Syatat Software Inc., San Jose, CA, USA) was used in the statistical process of fitting k.

## 3. Results and Discussion

### 3.1. Optimization of HPLC-MS/MS Conditions

**Figure 2:**
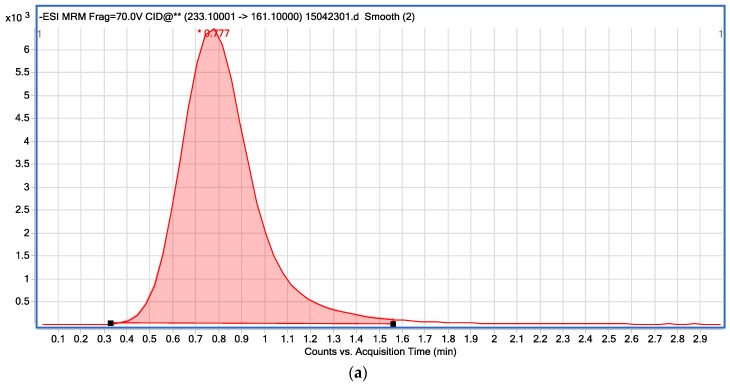

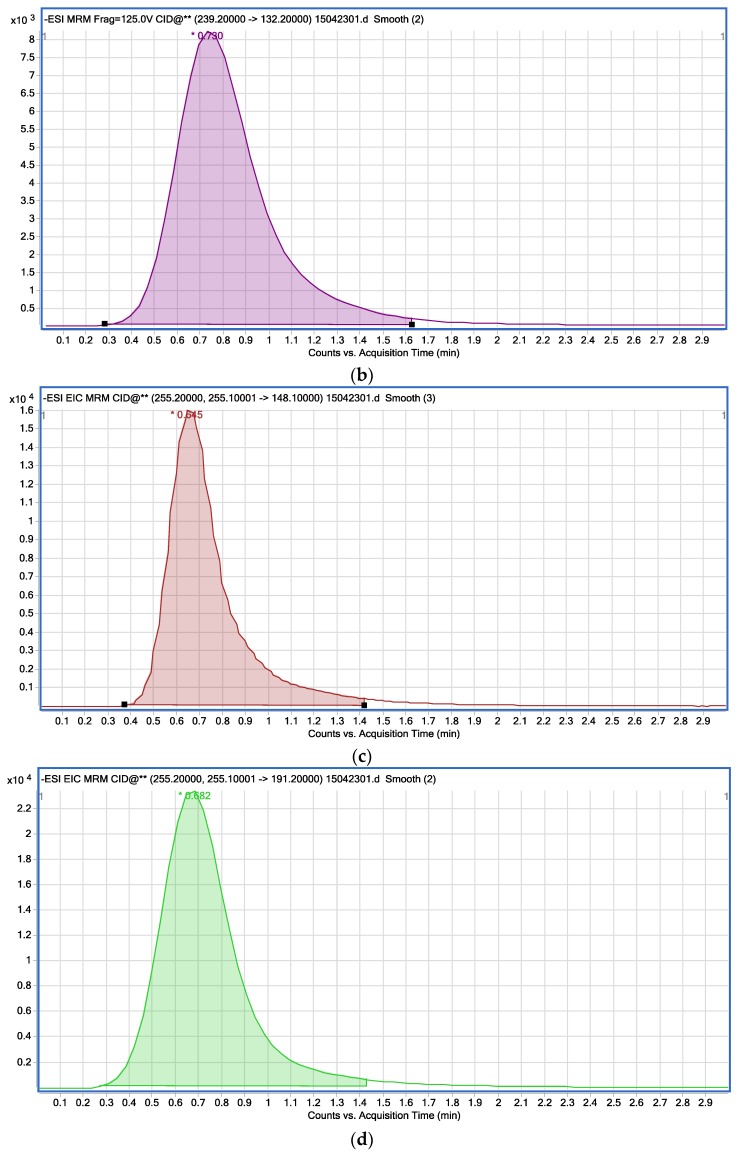
The LC-MS/MS chromatograph of (**a**) dichlorprop-P; (**b**) bentazone; (**c**) 6-hydroxy-bentazone, and; (**d**) 8-hydroxy-bentazone (mixed standard solution of 1 mg/L).

The composition of the mobile phase is an important parameter in adjusting retention time, selectivity, and peak shape in HPLC separation [29]. In this work, the acetonitrile-water and methanol-water mobile phase had been tested. Sharper peaks and better resolution were obtained using the acetonitrile-water system. In addition, different acetonitrile proportion (90%, 80%, 70%) had also been tested and, when the mobile phase was set at 90/10 (*v*/*v*) mixture of acetonitrile and water (containing 0.1% formic acid), a high response, well-shaped peaks and a short run time could be achieved, which was shown in Figure 2.

In this study, ESI in negative mode was selected since that four compounds are all acidic and easily lose H^+^. The [M−H]^+^ ion was chosen as a precursor ion for bentazone and its metabolites (6-hydroxy-bentazone and 8-hydroxy-bentazone) and [M−2H]^+^ was for dichlorprop-P because of its high relative intensity in a full scan, and then fragmentor of each precursor was optimized after running in SIM mode. After that, the product ions of each compound was selected and the collision energy for each ion transition was optimized. All compounds were analyzed in MRM mode (Table 2).

### 3.2. Optimization of Sample Pretreatment

One objective of our study was to establish a modified QuEChERS approach for the determination of dichlorprop-P, bentazone and its metabolites (6-hydroxy-bentazone and 8-hydroxy-bentazone) in wheat. The QuEChERS method, including solvent extraction, salting out, liquid-liquid partitioning, and d-SPE cleanup, has become a generic sample preparation technique for a variety of applications in pesticide residue analysis in the past several years [30]. In this work, different parameters affect the QuEChERS method, such as sample weight, extracting solvent and cleanup sorbent. A different sample weight (2.0 g, 5.0 g, 10.0 g) had also been tested and when grains of wheat was 5.0 g, wheat plants and wheat straw 2.0 g, satisfactory results could be achieved. Acetonitrile or ethyl acetate is often used as the extraction solvent for multi-residue analysis [22,31,32]. According to physicochemical properties of four analytes, acetonitrile was chosen as the extraction solvent. Besides that, to develop a reliable multi-residue method that can sufficiently extract acidic pesticides from cereals, the pH of the extraction is also important and should be suitably controlled. There are many published methods [33,34,35] for the analysis of acidic herbicides in cereals and related matrices recommended extraction with acidified solvent because acidic herbicides were more stable at low pH. Other authors reported that extract acidic herbicides with alkaline hydrolysis which could break up any covalent bond between the matrix components and acidic pesticides [36]. In this work, a simple study was conducted to compare formic acid water with ammonia water. The results showed that there was no significant difference for the recovery of dichlorprop-P, while low recoveries (˂70%) of bentazone, 6-hydroxy-bentazone and 8-hydroxy-bentazone were obtained when adding ammonia water to samples. Thus, formic acid water was chosen in this step. Then, NaCl was added and vortex extracted for 2 min, which was satisfied for the extraction of all the target compounds in three different kinds of matrices.

In the following cleanup procedure, d-SPE was adopted, which was a quick and easy method used in the QuEChERS procedure [37]. Three adsorbents bondesil-primary secondary amine (PSA), C_18_ and graphitized carbon black (GCB) were considered in this study. PSA is a weak anion exchanger which can remove various organic acids and fatty acids, as well as the target acidic pesticides. Therefore, PSA could not be chosen as an adsorbent to clean up the four compounds. Cleanup efficiencies of 50 mg C_18_ and 100 mg MgSO_4_, 50 mg GCB and 100 mg MgSO_4_, 100 mg MgSO_4_ for 1 mL of mixed standard solution of wheat plants (0.1 mg/L) were tested, respectively. After being vortexed for 30 s, the recoveries were as follows: for bentazone they were 41.4%, 67.7% and 84.9%; for 6-hydroxy-bentazone they were 45.7%, 47.5% and 100.2%; for 8-hydroxy-bentazone they were 45.1%, 32.9% and 91.5%, and; for dichlorprop-P they were 81.4%, 31.3% and 79.6%. This proved that C_18_ has some adsorption for bentazone and that GCB significantly adsorbs the four target compounds. Satisfactory recovery results and well-shaped peaks were obtained when 100 mg MgSO_4_ was used. Thus, only 100 mg MgSO_4_ was used for cleanup in this work.

### 3.3. Method Validation

#### 3.3.1. Calibration, LOD and LOQ

In this study, the matrix-matched calibration method was used to avoid possible matrix effects. Known pesticide amounts were added to the three representative extracts from control plots (grains of wheat, wheat plants and wheat straw) to obtain the final working matrix standard solution. The linearity for dichlorprop-P was studied in the range of 0.01–2.5 mg/kg in grains of wheat, 0.02–2.0 mg/kg in wheat plants and wheat straw; simultaneously, bentazone and its metabolites was 0.005–1.0 mg/kg in grains of wheat, 0.002–0.2 mg/kg in wheat plants and wheat straw. Good linearity was obtained with the correlation coefficient (R^2^) higher than 0.99.

The limit of detection (LOD) and the limit of quantification (LOQ) were defined as the concentration with a signal-to-noise ratio (S/N) of 3 and 10 [22]. As shown in Table 3, the LODs of the target compounds ranged from 0.8 μg/kg to 4 μg/kg and the LOQs were 2.5–12 μg/kg.

#### 3.3.2. Accuracy

Recovery studies were performed to evaluate the accuracy of the proposed by spiking the blank samples at three different concentration levels. Each spiking level was repeated five times for the calculation of RSD (relative standard deviation), which were used to show the precision of the method [38]. The EU SANTE/11945/2015 guidance document stipulates the average recovery in the range 70%–120%, with RSD less or equal 20% per each spiking level as the acceptance criteria for the validation of pesticide residue analytical methods [39]. As shown in Table 4, the mean recoveries of dichlorprop-P, bentazone, 6-hydroxy-bentazone and 8-hydroxy-bentazone in the range of 72.9%–107.4%, 76.1%–108.7%, 74.8%–94.0% and 75.2%–107.4%, with RSDs ranging from 2.5% to 11.4%, 1.0% to 10.7%, 1.3% to 9.2% and 1.6% to 8.0%, respectively, indicating good accuracy and precision of this approach.

### 3.4. Dissipation of Dichlorprop-P and Bentazone in Wheat Plants

**Figure 3:**
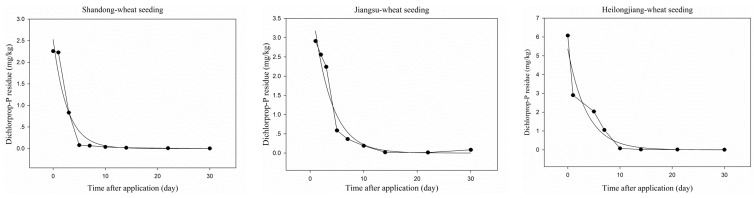
Dissipation of dichlorprop-P in wheat plants in Shandong, Jiangsu and Heilongjiang.

**Figure 4:**
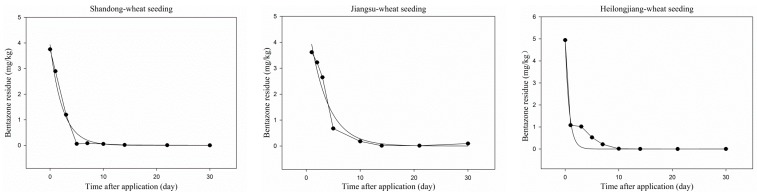
Dissipation of bentazone in wheat plants in Shandong, Jiangsu and Heilongjiang.

Figure 3 and Figure 4 showed the dissipation trends of dichlorprop-P and bentazone in wheat plants at different locations. As shown in Figure 3, the initial concentrations of dichlorprop-P in wheat plants were 2.26, 2.40 and 6.07 mg/kg in Shandong, Jiangsu and Heilongjiang. The difference in initial concentrations may be caused by different planting densities at the three sites or uneven spraying. At 30 days after application, the concentrations of dichlorprop-P were all reduced by more than 97% with the residue all ˂0.1 mg/kg (the lowest spiking level concentration) at the three sites. The dissipation dynamics of dichlorprop-P could be described by the first-order kinetics equation C = 2.5275e^−0.3690t^ (Shandong), C = 4.2685e^−0.2958t^ (Jiangsu) and C = 5.3620e^−0.2723t^ (Heilongjiang) with correlation coefficients (R^2^) of 0.9709, 0.9768 and 0.9604, respectively. The climates of the three experiment locations are different, but the *t*_1/2_ of dichlorprop-P were similar (1.9, 2.3 and 2.5 days at Shandong, Jiangsu and Heilongjiang, respectively), which showed that the dissipation of dichlorprop-P in wheat plant is not affected very much by the weather.

In Figure 4, the dissipation of bentazone in wheat plants is presented. The initial concentrations of bentazone in wheat plants were 3.75, 3.37 and 4.95 mg/kg at Shandong, Jiangsu and Heilongjiang. The difference from dichlorprop-P is that bentazone demonstrated a faster rate of dissipation. At 14 days after application, the concentrations of bentazone were all reduced by more than 97% at the three sites. At 30 days, bentazone residues were both ˂0.01 mg/kg (the lowest spiking level concentration) in Shandong and Heilongjiang and 0.09 mg/kg in Jiangsu. The fist-order kinetics equation of bentazone were C = 3.9357e^−0.4241t^ (Shandong), C = 5.2689e^−0.2942t^ (Jiangsu) and C = 4.9098e^−1.3394t^ (Heilongjiang) with correlation coefficients (R^2^) of 0.9900, 0.9791 and 0.9692, respectively. The dissipation half-life of bentazone in wheat plants calculated from the regression equation was 1.6, 2.4 and 0.5 in Shandong, Jiangsu and Heilongjiang, respectively, which showed that the dissipation of bentazone in wheat plants is possibly affected by differences in weather conditions among sites.

### 3.5. Terminal Residue of Four Analytes in Wheat Straw and Grain of Wheat

The terminal residue of four analytes applied at the two dosage levels (2547 g a.i./ha and 3820.5 g a.i./ha) were all below their lowest spiking level concentration. The results indicated that the terminal residue of dichlorprop-P in wheat was far below the MRLs (0.2 mg/kg established by EU) and bentazone (sum of bentazone, 6-hydroxy-bentazone and 8-hydroxy-bentazone) was below 0.1 mg/kg (established by EU, CAC, China). The residue data suggests the safe application of dichlorprop-P·bentazone at the recommended dosage to protect wheat from broadleaf weeds and nutgrass flatsedge.

## 4. Conclusions

In this work, a sensitive and fast analytical method based on QuEChERS and HPLC-MS/MS for the simultaneous determination of dichlorprop-P, bentazone, 6-hydroxy-bentazone and 8-hydroxy-bentazone in grains of wheat, wheat plants and wheat straw was developed and validated. With respect to linearity, recovery, sensitivity and repeatability, the developed method showed satisfactory validation results. This method was used to study dichlorprop-P and bentazone dissipation in wheat plants, as well as four analytes residues in grains of wheat and wheat straw. The results showed that the dissipation of dichlorprop-P and bentazone in wheat plants was fast, with half-lives of 1.9–2.5 days and 0.5–2.4 days, respectively. The final residues in grains of wheat and wheat straw were all lower than the MRL of four analytes.

## Figures and Tables

**Table 1:** Design of the field experiments for dichlorprop-P and bentazone residue and dissipation in wheat (grains of wheat, wheat plants and wheat straw).

Treatments		Dosage of Application (g a.i./ha)	Times of Application	Experiments	Days after the Last Application
Serial number	Areas (m^2^)				
1	30 × 3	2547	1	Grains of wheat/wheat straw residue	Harvest
2	30 × 3	3280.5	1	Grains of wheat/wheat straw residue	Harvest
3	30 × 3	3280.5	1	Dissipation in wheat plants	2 h, 1 day, 2 days, 3 days, 5 days, 7 days, 10 days, 14 days, 21 days, 30 days
4	30	0	-	Control treatment	Before harvest and harvest

**Table 2:** HPLC-MS/MS parameters of dichlorprop-P, bentazone, 6-hydroxy-bentazone and 8-hydroxy-bentazone.

Compound	*t*_R_ (min)	High-Performance Liquid Chromatography Coupled with Tandem Mass Spectrometry (HPLC-MS/MS)				
		Quantification Ion Transition	Collision Energy (V)	Confirmatory Ion Transition	Collision Energy (V)	Fragmentor (V)
Dichlorprop-P	0.74	233.1–161.1	5	233.1–125.2	25	70
Bentazone	0.70	239.2–132.2	20	239.2–197.1	15	125
				239.2–175.2	15	
6-hydroxy-bentazone	0.64	255.2–148.1	20	255.2–213.2	15	130
				255.2–121.1	25	
8-hydroxy-bentazone	0.65	255.1–191.2	10	255.1–148.1	20	120
				255.1–106.2	20	

**Table 3:** The calibration curves, coefficient of determination (R^2^) matrix effect and the limit of detection (LOD)/limit of quantification (LOQ) of dichlorprop-P, bentazone, 6-hydroxy-bentazone and 8-hydroxy-bentazone in wheat (grains of wheat, wheat plants and wheat straw).

Compound	Matrix	Matrix-Matched Calibration Curve	R^2^	LOD (μg/kg)	LOQ (μg/kg)
Dichlorprop-P	Grains of wheat	Y = 30939X − 1515.9	0.9911	2	7
	Wheat plants	Y = 32278X−486.86	0.9995	4	12
	Wheat straw	Y = 23968X + 196.11	0.9999	2	7
Bentazone	Grains of wheat	Y = 89621X − 1369.4	0.9959	1	3
	Wheat plants	Y = 77068X + 12.216	0.9997	3	9
	Wheat straw	Y = 48882X − 95.561	0.9995	3	9
6-hydroxy-bentazone	Grains of wheat	Y = 150628X − 731.53	0.9989	2	7
	Wheat plants	Y = 144706X − 80.588	0.9998	1	3
	Wheat straw	Y = 124585X − 178.16	0.9999	0.8	2.5
8-hydroxy-bentazone	Grains of wheat	Y = 309797X + 853.47	0.9996	2	6
	Wheat plants	Y = 360626X − 477.38	0.9995	3	9
	Wheat straw	Y = 264981X − 168.88	0.9996	1	4

**Table 4:** Recoveries (*n* = 5) and relative standard deviations (RSDs) of dichlorprop-P, bentazone, 6-hydroxy-bentazone and 8-hydroxy-bentazone in wheat (grains of wheat, wheat plants and wheat straw).

Compound	Matrix	Fortified Level (mg/kg)	Average Recovery (%)	RSD (%)
Dichlorprop-P	Grains of wheat	0.1	85.9	7.0
		1	85.0	5.7
		5	72.9	4.0
	Wheat plants	0.1	82.0	6.3
		1	96.1	4.0
		5	82.9	11.4
	Wheat straw	0.1	107.4	8.0
		1	82.1	5.1
		5	98.6	2.5
Bentazone	Grains of wheat	0.01	84.6	7.6
		0.1	93.6	3.1
		0.5	76.1	1.0
	Wheat plants	0.01	82.0	10.7
		0.1	96.1	3.8
		0.5	82.9	3.7
	Wheat straw	0.01	108.7	9.6
		0.1	96.2	2.2
		0.5	87.0	3.4
6-hydroxy-bentazone	Grains of wheat	0.01	92.9	2.9
		0.1	81.4	9.2
		0.5	84.7	1.3
	Wheat plants	0.01	90.8	1.4
		0.1	94.0	4.3
		0.5	74.8	4.2
	Wheat straw	0.01	87.5	5.8
		0.1	89.7	3.3
		0.5	84.9	3.5
8-hydroxy-bentazone	Grains of wheat	0.01	86.8	5.4
		0.1	88.9	7.1
		0.5	79.5	6.3
	Wheat plants	0.01	85.0	7.6
		0.1	95.9	4.5
		0.5	75.2	5.7
	Wheat straw	0.01	107.4	8.0
		0.1	82.1	5.1
		0.5	84.5	1.6

## References

[1] He J., Shen Y., Wang D., Sun X., Fan M., Liu X. (2010). Dissipation and residues of 2,4-d-dimethylammonium in wheat and soil. Bull. Environ. Contam. Toxicol..

[2] Tu H. (2001). Development in research and controlling of weeds in fields of China. Nongyao.

[3] Ma S.Y., Kim S.W., Chun J.C. (1998). Antagonistic mode of action of fenoxaprop-p-ethyl phytotoxicity with bentazon. Korean J. Weed Sci..

[4] Food and Agriculture Organization of the United Nations. http://www.fao.org/fileadmin/templates/agphome/documents/Pests_Pesticides/JMPR/Reports_1991-2006/REPORT1991.pdf.

[5] Codex Alimentarius Commission. ftp://ftp.fao.org/codex/meetings/CCPR/CCPR46/pr46_02e.pdf.

[6] EU Pesticides Database. http://ec.europa.eu/food/plant/pesticides/eu-pesticides-database/public/?event=pesticide.residue.CurrentMRL&language=EN.

[7] China Pesticide Information Network. http://202.127.42.84/tbt-sps/mrlsdb/queryMrlsdb.do.

[8] Pulgarín J.A.M., Bermejo L.F.G., Rodríguez S.B. (2015). Direct determination of dichlorprop in commercial formulations, tomato and fruit samples using photochemically induced fluorescence. Food Anal. Methods.

[9] You X., Liang L., Liu F. (2014). Dissipation and residues of clethodim and its oxidation metabolites in a rape-field ecosystem using QuEChERS and liquid chromatography/tandem mass spectrometry. Food Chem..

[10] Sanchis-Mallols J.M., Sagrado S., Medina-Hernandez M.J., Camanas R.M.V., Bonet-Domingo E. (1998). Determination of phenoxy acid herbicides in drinking waters by HPLC and solid phase extraction. J. Liq. Chromatogr. Relat. Technol..

[11] Tsipi D., Hiskiai A., Heberer T., Stan H.-J. (1998). Determination of acidic pesticides in the drinking water of Greece using capillary gas chromatography-mass spectrometry. Water Air Soil Pollut..

[12] Vink M., van der Poll J.M. (1996). Gas chromatographic determination of acid herbicides in surface water samples with electron-capture detection and mass spectrometric confirmation. J. Chromatogr. A.

[13] Meng C.-K., Werner S.L., Furlong E.T. (2008). Determination of pesticide residue in water under positive and negative ion mode by solid-phase extraction and LC/MS/MS. Huanjing Huaxue.

[14] Thorstensen C.W., Lode O., Christiansen A.L. (2000). Development of a solid-phase extraction method for phenoxy acids and bentazone in water and comparison to a liquid-liquid extraction method. J. Agric. Food Chem..

[15] Nishina T., Murakawa H., Fukushima K., Tobino T. (2006). Pesticide residue monitoring method in agricultural products with GC/MS and LC/MS/MS. Ann. Rep. Kumamoto Prefect. Inst. Public Health Environ. Sci..

[16] Paul N.T., Mark D.S., John N.L. (1998). Supercritical fluid extraction of acidic herbicides from sediment. Int. J. Environ. Anal. Chem..

[17] Jin H., Wang Y., Lan J., Ma S. (2012). Determination of 192 pesticides in Flos Lonicerae by gas chromatography-mass spectrometry. Chin. Pharm. J..

[18] Zhang L., Gui J.-Y., Zhang Y.-T., Zuo H.-Y., Li X.-Y., Zhang G.-Q. (2011). GC-MS determination of acidic herbicides in water with prederivatization. Physic. Test. Chem. Anal..

[19] Reddersen K., Heberer T. (2003). Multi-compound methods for the detection of pharmaceutical residues in various waters applying solid phase extraction (SPE) and gas chromatography with mass spectrometric (GC-MS) detection. J. Sep. Sci..

[20] Yan H., Huang Z., Zhang Y., Li Y., Wang M. (2009). Determination of 29 acidic herbicide residues in tea by gas chromatography-mass spectrometry. SEPU.

[21] Dong Z., Zhao S., Wei F., Jiang J., Jiang W., Xie B. (2005). Determination of herbicide residues in rice by GC-ECD with methylation derivatization. Chin. J. Public Health.

[22] Anastassiades M., Lehotay S.J., Stajnbaher D., Schenck F.J. (2003). Fast and easy multiresidue method employing acetonitrile extraction/partitioning and “dispersive solid-phase extraction” for the determination of pesticide residues in produce. J. AOAC Int..

[23] Hou X., Han M., Dai X.H., Yang X.F., Yi S. (2013). A multi-residue method for the determination of 124 pesticides in rice by modified QuEChERS extraction and gas chromatography–tandem mass spectrometry. Food Chem..

[24] Guan W., Xu P., Wang K., Song Y., Zhang H. (2011). Determination and study on dissipation of 1-naphthylacetic acid in garlic and soil using high performance liquid chromatography–tandem mass spectrometry. Food Chem. Toxicol..

[25] Cunha S.C., Lehotay S.J., Mastovska K., Fernes J.O., Beatriz M., Oliveira P.P. (2007). Evaluation of the QuEChERS sample preparation approach for the analysis of pesticide residues in olives. J. Sep. Sci..

[26] Lehotay S.J., Mastovská K., Yun S.J. (2005). Evaluation of two fast and easy methods for pesticide residue analysis in fatty food matrixes. J. AOAC Int..

[27] Omirou M., Vryzas Z., Papadopoulou-Mourkidou E., Economou A. (2009). Dissipation rates of iprodione and thiacloprid during tomato production in greenhouse. Food Chem..

[28] Rajib K., Gita K. (2009). Persistence, metabolism and safety evaluation of thiamethoxam in tomato crop. Pest Manag. Sci..

[29] Lu Q., Chen L., Lu M. (2010). Extraction and analysis of auxins in plants using dispersive liquid-liquid microextraction followed by high-performance liquid chromatography with fluorescence detection. J. Agric. Food Chem..

[30] Walorczyk S. (2014). Validation and use of a QuEChERS-based gas chromatographic–tandem mass spectrometric method for multiresidue pesticide analysis in blackcurrants including studies of matrix effects and estimation of measurement uncertainty. Talanta.

[31] Ferrer I., Thurman E.M. (2008). Multi-residue method for the analysis of 101 pesticides and their degradates in food and water samples by liquid chromatography/time-of-flight mass spectrometry. J. Chromatogr. A.

[32] Jian P., Xia X.X., Liang J. (2008). Analysis of pesticide multi-residues in leafy vegetables by ultrasonic solvent extraction and liquid chromatography-tandem mass spectrometry. Ultrason. Sonochem..

[33] Sack C., Vonderbrink J., Smoker M., Smith R.E. (2015). Determination of acid herbicides using modified quechers with fast switching ESI+/ESI−LC-MS/MS. J. Agric. Food Chem..

[34] Shida S.S., Nemoto S., Matsuda R. (2015). Simultaneous determination of acidic pesticides in vegetables and fruits by liquid chromatography–tandem mass spectrometry. J. Environ. Sci. Health B.

[35] Koesukwiwat U., Sanguankaew K., Leepipatpiboon N. (2008). Rapid determination of phenoxy acid residues in rice by modified quechers extraction and liquid chromatography–tandem mass spectrometry. Anal. Chim. Acta.

[36] Santilio A., Stefanelli P., Girolimetti S., Dommarco R. (2011). Determination of acidic herbicides in cereals by quechers extraction and LC/MS/MS. J. Environ. Sci. Health B.

[37] Lehotay S.J. (2007). Determination of pesticide residues in foods by acetonitrile extraction and partitioning with magnesium sulfate: Collaborative study. J. AOAC Int..

[38] Ferrer C., Martínez-Bueno M.J., Lozano A., Fernández-Alba A.R. (2011). Pesticide residue analysis of fruit juices by LC-MS/MS direct injection. One year pilot survey. Talanta.

[39] European Commission Guidance Document on Analytical Quality Control and Method Validation Procedures for Pesticides Residues Analysis in Food and Feed. http://ec.europa.eu/food/plant/docs/plant_pesticides_mrl_guidelines_wrkdoc_11945_en.pdf.

